# Serum hCG levels in the prediction of molar pregnancy below 11 weeks of gestational age

**DOI:** 10.1590/1806-9282.20240659

**Published:** 2024-12-16

**Authors:** Lucas Ribeiro Borges de Carvalho, Rafaela Tessaro de Assis, Antônio Braga, Tatiana Carvalho de Souza Bonetti, Edward Araujo, Rosiane Mattar, Sue Yazaki Sun

**Affiliations:** 1 Universidade Federal de São Paulo, Escola Paulista de Medicina, Department of Obstetrics – São Paulo (SP), Brazil.; 2 Universidade Federal do Rio de Janeiro, Department of Obstetrics and Gynecology – Rio de Janeiro (RJ), Brazil.; 3 Universidade Federal de São Paulo, Escola Paulista de Medicina, Department of Gynecology – São Paulo (SP), Brazil.

**Keywords:** Miscarriage, Complete hydatidiform mole, Chorionic gonadotropin, First trimester pregnancy

## Abstract

**OBJECTIVE:**

The aim of this study was to evaluate the serum hCG level in the differential diagnosis between non-molar miscarriage and complete hydatidiform mole in<11 weeks gestation.

**METHODS:**

This was a retrospective collaborative cohort study. This study included women with gestational age<11 weeks, with ultrasound evidence of failed pregnancy and available serum hCG pre-uterine evacuation, divided into two groups: the non-molar miscarriage group and the complete hydatidiform mole group. Serum hCG levels were compared according to gestational age. Statistical analysis used a nonparametric test with a 5% significance level (p<0.05).

**RESULTS:**

In total, 416 patients were included, out of which 79 were included in the non-molar miscarriage group and 337 in the complete hydatidiform mole group. The calculated power of the sample was more than 80%. Data analysis showed that the 75th quartile of the median in the non-molar miscarriage group was always lower than the 25th quartile of the median in the complete hydatidiform mole group [9,721 mUI/mL/16,435 mUI/mL (6–7 weeks), 20,229 mUI/mL/64,911 mUI/mL (8–9 weeks), and 29,633 mUI/mL/126,278 mUI/mL (10–11 weeks), respectively; p<0.001].

**CONCLUSION:**

Facing failed pregnancies, hCG>16,435 mUI/mL at 6–7 weeks, hCG>64,911 mUI/mL at 8–9 weeks, and hCG >126,278 mUI/mL at 10–11 weeks were most prevalent on complete hydatidiform mole diagnosis. On the contrary, hCG<30,000 mUI/mL at 10–11 weeks was most prevalent in non-molar miscarriage diagnosis.

## INTRODUCTION

Vaginal bleeding is one of the most common complaints in the first trimester of pregnancy and can indicate imminent miscarriage, molar pregnancy, and ectopic pregnancy^1^. Molar pregnancies, known as partial hydatidiform mole (PHM) and complete hydatidiform mole (CHM), represent the benign spectrum of trophoblastic disorders of pregnancy. These early gestational pregnancies are difficult to distinguish from missed miscarriages, both clinically and on ultrasound^2,3^. Early diagnosis and correct treatment of molar pregnancy are necessary, as prolonged molar pregnancy can lead to severe maternal morbidity due to hemorrhage, hyperemesis, pre-eclampsia, respiratory failure, and thyrotoxic crisis^4,5^. In CHM, typical sonographic features of sonolucent cystic areas of variable size without embryo or embryonic structures occupying the uterine cavity are seen at 10–12 weeks^6,7^. However, in early gestational age, the sonographic features have been described as nonspecific and cannot be distinguished from incomplete or missed abortions^8^.

In normal pregnancy, serum hCG levels increase exponentially during the first trimester, reaching higher levels between 11 and 13 weeks of gestation. In contrast, this increase does not occur in failed pregnancies^9^. In molar pregnancies, trophoblastic hyperplasia results in higher serum hCG levels than in normal pregnancies of the same gestational age^10^. Considering that the ultrasound images of non-molar miscarriage (NMM) and early CHM are confusing, and knowing that hCG in CHM is higher than in normal pregnancies, evaluating the level of hCG could be a predictor of CHM<11 weeks of gestation in non-evolving pregnancies^11,12^.

Therefore, the aim of this study was to evaluate the serum hCG level in the differential diagnosis between NMM and CHM at<11 weeks of gestation. This hCG level may be useful as a tool to avoid inappropriate prolongation of CHM.

## METHODS

### Research type

This was a retrospective collaborative cohort study conducted at the São Paulo Hospital Trophoblastic Disease Center of the Federal University of São Paulo and the Maternity Hospital Trophoblastic Disease Center of the Federal University of Rio de Janeiro, approved by both Institutional Review Boards under numbers CAAE 13739719.8.0000.5505 and 13739719.8.3001.5275, respectively.

### Setting and sample

It used a non-probabilistic convenience sample, which included patients treated from January 1, 2009, to December 31, 2018, registered in the Luiz Camano Trophoblastic Disease Register of the São Paulo Hospital Trophoblastic Disease Center and Maternity Hospital Trophoblastic Disease Center Register of the Federal University of Rio de Janeiro. Data were collected from December 1, 2019, to March 31, 2021.

Included were all patients aged 12–55 years, with a gestational age of<11 weeks (calculated to the last menstrual period), an ultrasound of failed pregnancy or suggestive of molar pregnancy, serum hCG level available before evacuation, and histopathologic diagnosis of CHM according to Szulman and Surti^13^, confirmed by negative p57 immunohistochemical examination, or products of conception (POC). Serum hCG was measured by the chemiluminescence method (using the Immulite^®^ Siemens and Architect^®^ Abbot commercial assays). We excluded patients with incomplete medical records and inconclusive histopathologic diagnosis.

The patients were divided into two groups: the CHM group and the NMM group. The CHM group included patients with histopathologic diagnoses of CHM. The NMM group included patients with histopathologic diagnosis of POC without molar features. In each group, women were classified according to gestational age at 6–7 weeks, 8–9 weeks, and 10–11 weeks of gestation. We retrieved the following data from the patients’ medical records: maternal age, ethnicity, educational level, number of pregnancies and parity, gestational age (calculated to the last menstrual period), and serum hCG level from 7 to 0 days before the diagnosis of failed or molar pregnancy.

Serum hCG levels were compared between CHM and NMM according to gestational age on the day of evacuation.

### Statistical analysis

The distribution of hCG values was checked by the Shapiro-Wilk test, which indicated a non-normal distribution (<0.001). Therefore, continuous numerical variables were presented as median and interquartile range (IQR 25–75), and categorical variables were presented as frequencies and percentages. Statistical analysis was performed using a nonparametric test, 5% significance level (p<0.05), and SPSS 21 software (IBM, Armonk, NY, USA).

The calculated power of the sample was higher than 80% (86.1% for 6–7 weeks comparison, 99.6% for 8–9 weeks, and 99.9% for 10–11 weeks).

## RESULTS

**Figure 1:**
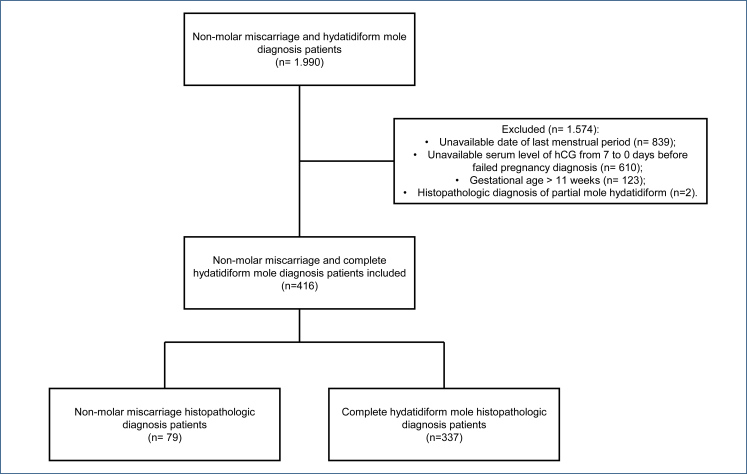
Flowchart of patients treated from January 1, 2009, to December 31, 2018, included in the study.

The study included 416 patients, 79 from the NMM group and 337 from the CHM group. This study analyzed 1,990 medical records, but 1,574 were excluded because 839 patients did not have the date of their last menstrual period available, 610 did not have the serum level of hCG from 7 to 0 days before failed pregnancy diagnosis available, 123 had gestational age>11 weeks of gestation, and two patients had a histopathologic diagnosis of PHM (Figure 1).

The median age of patients and pregnancies were similar: 29 and 27, despite parity being statistically different (1 and 0, respectively, in the NMM and CHM groups), which had no clinical significance in the NMM and CHM groups. The median number of pregnancies was two in both groups. In terms of race, brown and white patients predominated in the NMM and CHM groups, respectively. The median serum hCG levels were 3.738 and 100.150 mUI/mL at 6–7 weeks of gestation, 8.669 and 136.213 mUI/mL at 8–9 weeks of gestation, and 8.554 and 207.806 mUI/mL at 10–11 weeks of gestation in the NMM and CHM groups, respectively (p<0.001) (Table 1).

**Table 1:** Characteristics of patients from non-molar miscarriages group and complete hydatidiform mole group.

Characteristics	NMM				CHM				p-value
	n	Minimum–maximum	Median (IQR 25–75)	Mean±SD	N	Minimum–maximum	Median (IQR 25–75)	Mean±SD	
Age	79	15–43	29 (23.5–34)	28.6±6.9	337	13–52	27 (22–34)	28.4±8.6	0.488
Pregnancies	79	1–6	2 (1–3)	2.3±1.2	337	1–10	2 (1–3)	2.2±1.6	0.105
Parity	79	0–4	1 (0–1.5)	1.0±1.0	337	0–8	0 (0–1)	0.8±1.2	0.047
Ethnicity	n		Percentage		n		Percentage		p
White	12		36.40%		173		55.30%		0.182
Black	6		18.20%		32		10.20%		
Mixed	14		42.40%		103		32.90%		
Other	1		3.00%		5		1.60%		
Not informed	46		–		24		–		–

NMM: non-molar miscarriages; CHM: complete hydatidiform mole; IQR: interquartile range; SD: standard deviation. Categorical variables were compared by chi-square and numerical variables by Mann-Whitney test. Statistical analysis of ethnicity was performed differently due to small sample sizes.

The 75th quartile of the median serum hCG level was 9,721, 20,229, and 29,633 mUI/mL at 6–7, 8–9, and 10–11 weeks of gestation, respectively, in the NMM group. On the contrary, the 25th quartile of the median serum hCG level was 16,435, 64,911, and 126,278 mUI/mL in the 6–7, 8–9, and 10–11 weeks gestations in the CHM group, respectively (Table 2).

**Table 2:** Comparative analysis of serum hCG values in the non-molar miscarriages group and complete hydatidiform mole group, according to gestational age at uterine evacuation day.

GA	NMM group (mUI/mL)				CHM group (mUI/mL)				Mann-Whitney and Shapiro-Wilk test
	n	Mean±SD	Median (IQR 25–75)	Min–Max	N	Mean±SD	Median (IQR 25–75)	Min–Max	p-value
6–7 weeks	19	11,002±16,678	3,738 (531–9,721)	51–57,075	47	198,866±245,453	100,150 (16,435–236,965)	924–982,174	<0.001
8–9 weeks	29	16,075±18,904	8,669 (4,684–20,229)	737–73,489	120	209,270±222,869	136,213 (64,911–282,009)	8,573–999,000	<0.001
10–11 weeks	31	20,620±27,986	8,554 (3,340–29,633)	171–123,941	170	292,585±255,171	207,806 (126,278–395,340)	3,422–999,000	<0.001

GA: gestational age; NMM: non-molar miscarriages; CHM: complete hydatidiform mole; IRQ: interquartile range; SD: standard deviation.

The serum hCG level progression showed a significant increase over time in the CHM group, with an increase of 100,000 mUI/mL from 8–9 to 10–11 weeks of pregnancy. Alternatively, when it comes to the NMM group, the serum hCG level changed almost nothing from 8–9 to 10–11 weeks of gestation.

## DISCUSSION

Based on our data, it is possible to highlight the importance of the serum hCG level in the differential diagnosis of NMM and CHM in the first trimester of pregnancy, in cases of failed pregnancy, especially because when analyzing the median of both groups, the 75th quartile of the median in the NMM group is always lower than the 25th quartile of the CHM group.

The gold standard for diagnosis of CHM is a histopathologic examination of material obtained from uterine evacuation. However, at early gestational ages of 6–9 weeks, the macroscopic and microscopic characteristics of CHM do not always allow it to be distinguished from PHM and NMM^14^. Therefore, when uterine evacuation is performed at these gestational ages due to the aforementioned hCG levels, histopathologic examination should be performed by pathologists experienced in placental diseases and with immunohistochemical study of p57 protein for better diagnostic differentiation^15,16^. In CHM, p57 expression will be negative in the cytotrophoblast and villous stromal cells because it is an androgenetic conception, i.e., it has no maternal genetic material^15,16^. It is important to know how to differentiate between NMM and CHM in cases of failed intrauterine pregnancy because the behavior is completely different. In NMM cases, the behavior is expectant^17,18^, whereas in CHM cases, the behavior is early evacuation to avoid complications such as hemorrhage, hyperemesis, preeclampsia, hyperthyroidism, respiratory failure, and maternal death^19–21^. In cases of early gestational age, vaginal ultrasound does not have the usual characteristics; therefore, hCG plays an important role in determining the management^7^.

In our study, the 75th quartile of the NMM group and the 25th quartile of the CHM group were 9,721 mUI/mL and 16,435 mUI/mL (6–7 weeks) and 20,229 mUI/mL and 64,911 mUI/mL (8–9 weeks), respectively. Therefore, it can be concluded that in the face of failed pregnancies, serum hCG levels of 16,435 mUI/mL at 6–7 weeks and 64,911 mUI/mL at 8–9 weeks of gestation suggest a diagnosis of CHM, and immediate uterine evacuation should be performed. In addition, the 75th quartile of the median of the NMM group and the 25th quartile of the CHM group at 10–11 weeks were 29,633 and 126,278 mU/mL, respectively, which is consistent with previously published studies showing that serum hCG levels above 100,000 mU/mL are suggestive of CHM in the second trimester of pregnancy^12,22^. Furthermore, in our data, the NMM group of 10–11 weeks had a median of 8,553 mUI/mL and a 75th quartile of 29,633 mUI/mL; therefore, we suggest that it is possible to adopt an expectant behavior if the hCG is below 30,000 mUI/mL, in 10–11 weeks of gestation.

In an analysis of previous literature on this topic, one study found hCG levels to be significantly higher in CHM than in NMM (10–200 MoM vs. 0.6 MoM, respectively), which is consistent with our findings. In this study, hCG levels were available in only 7 CHM and 18 NMM out of a total of 2,768 patients, preventing the authors from suggesting an accurate hCG level for clinical practice in miscarriage^6^.

Another study showed that median hCG levels are higher in CHM than in NMM (63,211 UI/L vs. 21,988 UI/L), similar to our findings^8^. In their sample, 2,398 women with NMM and 506 with CHM diagnoses had hCG levels available before evacuation. In our study, we believe that the higher median hCG level is due to the inclusion of 72 PHM cases. Both previous studies compared CHM with NMM when evaluating hCG levels without specifying the type of CHM and without stratifying for gestational age, unlike our study. Another comparison bias can be attributed to the difference in the tests used to detect hCG levels. In our study, we used Architect^®^ and Immulite^®^, while the second mentioned study used Access Immunoassay Systems.

Due to the retrospective nature of our study, and using data from two reference centers, pre-evacuation hCG levels were not available in 610 women because many referral centers do not have accurate quantitative hCG measurement techniques. Furthermore, in many cases<11 weeks of gestation, the diagnosis of CHM was made after uterine evacuation based on histopathologic findings^1,10,23^, and quantitative hCG levels were not requested at the time of first aid^5^.

The lack of pre-evacuation hCG levels in approximately 31% (610/1990) of women assisted during the study period represents an important limitation of our study, preventing the acquisition of a cut-off for predicting CHM at any gestational age. On the contrary, the hCG>64,911 mUI/mL at 8–9 weeks gestation, although not conclusive for MHC, is of particular interest for clinical practice as it suggests that the best course of action is uterine evacuation. The median hCG levels in all gestational age groups were>100,000 mUI/mL. Thus, finding this level of hCG also suggests that immediate uterine evacuation is the appropriate course of action. On the contrary, at 10–11 weeks gestational age, an hCG level <30,000 mUI/mL favors expectant management.

## CONCLUSION

In summary, facing failed pregnancies, hCG >16,435 mUI/mL at 6–7 weeks, hCG >64,911 mUI/mL at 8–9 weeks, and hCG >126,278 mUI/mL at 10–11 weeks were most prevalent on CHM diagnosis. On the contrary, hCG<30,000 mUI/mL at 10–11 weeks gestation was most prevalent in NMM diagnosis.
